# Hyperacute optic neuritis in a patient with COVID-19 infection and vaccination: a case report

**DOI:** 10.1186/s12886-023-02825-4

**Published:** 2023-02-28

**Authors:** Jessica Zhang, Devon Joiner, Cheng Zhang

**Affiliations:** 1 Albert Einstein College of Medicine, 111 East 210th Street, Room 306, Bronx, NY 10461 USA; 2 Department of Ophthalmology and Visual Sciences, Montefiore Medical Center, Albert Einstein College of Medicine, Bronx, NY USA

**Keywords:** Optic neuritis, Optic nerve, COVID-19, SARS-CoV-2

## Abstract

**Background:**

As scientific knowledge continues to grow regarding coronavirus disease 2019 (COVID-19) infection, several neuro-ophthalmological manifestations have emerged, including rare reports of optic neuritis. Optic neuritis is an inflammatory demyelinating condition of the optic nerve that typically presents as subacute, unilateral vision loss and pain on eye movement. Several cases of COVID-19 infection and COVID-19 vaccination related cases of optic neuritis have been reported. We present a case of hyperacute, unilateral optic neuritis after both recent COVID-19 infection and subsequent booster vaccination.

**Case presentation:**

Within two hours after receiving her COVID-19 booster vaccination, a 58-year-old female began experiencing bilateral eye pain, worsened by eye movements. The patient had previously contracted a mild COVID-19 infection three weeks prior to receiving her booster vaccination, confirmed by a rapid antigen test. The pain persisted in her right eye for a week at which time she presented to an ophthalmology clinic. She denied any changes to her visual acuity. Neuroimaging revealed right optic nerve enhancement, and the patient was admitted to the hospital for a course of intravenous steroids, which quickly resolved her eye pain.

**Conclusion:**

To our knowledge, this is the first reported case of COVID-19 related optic neuritis following both COVID-19 infection and vaccination. High clinical suspicion is needed to make the appropriate diagnosis, as cases of COVID-19 related optic neuritis may exhibit mild presentations, as was the case with our patient.

## Background

Since the first reported cases of severe acute respiratory syndrome coronavirus 2 (SARS-CoV-2) infection in December of 2019, the resulting coronavirus disease 2019 (COVID-19) pandemic has had devastating consequences for millions of people around the world, and has dramatically transformed society and healthcare systems. COVID-19 is well known to be a respiratory illness, with severe cases resulting in acute respiratory distress syndrome and other respiratory complications that have led to significant morbidity and mortality. As the COVID-19 pandemic continues to unfold and evolve, our knowledge of extra-pulmonary manifestations of COVID-19 continues to grow as well. We now know COVID-19 disease to be a profoundly hyperinflammatory condition [1], with a tendency toward hypercoagulability and autoimmune reactions [2, 3].

Numerous neurological sequalae of COVID-19 infection have since been discovered, including seizure, strokes, and rare cases of Guillain-Barré [4, 5]. Rare neuro-ophthalmologic complications, particularly optic neuritis, have been documented as well, including reports of both COVID-19 infection and COVID-19 vaccination-related cases [6–8]. We present here a case of hyperacute, unilateral optic neuritis with a mild clinical presentation in a patient who recently contracted COVID-19 and subsequently received her COVID-19 booster. To our knowledge, this is the first reported case of optic neuritis following both recent COVID-19 infection and COVID booster vaccination.

## Case presentation

In January of 2022, a 58-year-old female with no relevant past medical or ocular history began experiencing bilateral eye pain within two hours after receiving her Pfizer COVID booster vaccine. Three weeks prior, she had contracted a mild case of COVID-19, confirmed by a rapid COVID-19 antigen test, and by the time of her booster, had fully recovered. In addition to eye pain, the patient began experiencing flu-like symptoms the following day along with a right sided pressure-like headache. The pain resolved in the left eye after one day, but persisted in the right eye, and was characterised by severe pressure-like pain worsened by eye movement. She denied changes in visual acuity. After one week of no improvement, she presented to an ophthalmology clinic and was noted to have a mild left hypertropia on right gaze, a -3 deficit in supraduction on the right, and right eye pain, especially with upgaze. She otherwise had a visual acuity of 20/20 in both eyes, no afferent pupillary defect (APD), full colour vision, and a normal slit lamp examination. Corneal sensation was not assessed, but facial sensation (CNV_1_-V_3_) was grossly intact. On dilated fundus exam, the patient was noted to have mild nasal and inferior optic nerve rim elevation, but without disc swelling in the right eye. Optic coherence tomography (OCT) was obtained with normal thickness in both eyes, and a Humphrey visual field test (HVF) showed full visual fields bilaterally.

**Fig. 1:**
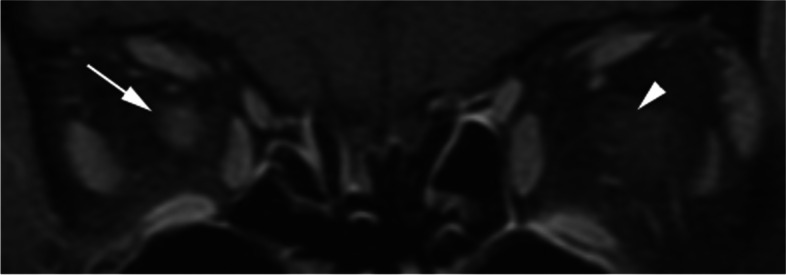
MRI orbits with contrast and fat suppression (coronal view) showing right optic nerve enhancement (arrow), with no enhancement in the left optic nerve (arrowhead)

At this point, a mild inflammatory process of the optic nerve was suspected, and the patient was sent to the emergency room to obtain magnetic resonance imaging (MRI) of the brain, orbits, and spine with contrast for further work-up. The MRI orbits revealed right optic nerve enhancement (Fig. 1). In addition, there were several nonspecific foci of high intensity in the periventricular and centrum semiovale regions of the brain that were suspected to be due to microvascular ischemic changes. MRI of the cervical, thoracic, and lumbar spine showed no evidence of abnormal cord signal or spinal canal enhancement. The patient was subsequently admitted to the hospital for further work-up and management, and she was started on a three-day course of intravenous methylprednisolone 250 mg every six hours for right mild optic neuritis. Her eye pain and motility deficits quickly improved on the steroid course, and she was discharged on an 11-day oral steroid taper. While the pain in the right eye resolved shortly after beginning her steroid course, the patient continued to endorse right eye discomfort for several weeks, which resolved over the next month.

A full laboratory work-up for the patient’s optic neuritis was also obtained, including antineutrophil cytoplasmic antibodies (ANCA), angiotensin-converting enzyme (ACE), antinuclear antibody (ANA), syphilis, human immunodeficiency virus (HIV), Lyme disease, tuberculosis, and erythrocyte sedimentation rate (ESR). In addition, she was also tested for neuromyelitis optica (NMO) and myelin oligodendrocyte glycoprotein (MOG) antibodies by cell-based assay, all of which were unremarkable. By the time of hospitalization, she was COVID-19 seronegative. After discharge from the hospital, the patient followed up outpatient with a neurologist at a specialised multiple sclerosis centre in the end of March 2022. Neurological exam was completely normal, and it was suspected that her optic neuritis was due to a post-vaccine or COVID-19-related demyelinating attack.

The patient followed up in an ophthalmology clinic in the beginning of May. At that time, she had no ocular complaints and her eye discomfort had completely resolved. On exam, her vision was stable at 20/25 in the right eye and 20/20 in the left eye, she had no APD, and her visual fields and extraocular movements were full. A repeat OCT and HVF were ordered and were found to be normal. The patient has repeat MRI brain and orbits scheduled in the upcoming months.

## Discussion and conclusions

Optic neuritis is an inflammatory demyelinating condition of the optic nerve typically characterised by subacute, unilateral decreased vision and periorbital pain worse with eye movement. Optic neuritis is often the first presenting sign of multiple sclerosis (MS), and thus warrants a thorough evaluation for MS including MRI brain and orbits with contrast as well as laboratory testing [9]. Less commonly, optic neuritis has been known to be a complication of both viral infection and vaccination. There have been reported cases of optic neuritis following influenza, mumps, varicella zoster, cytomegalovirus, and Epstein-Barr infection, as well as following inoculation with influenza, rabies, measles, mumps, rubella (MMR), hepatitis A and B vaccinations [10]. The pathophysiology is likely related to an autoimmune reaction triggered in patients with an underlying predisposition, rather than via direct bacterial or viral spread to the optic nerve [10].

Since the beginning of the COVID-19 pandemic, optic neuritis has been described in many cases of COVID-19 infection, as well as following several cases of COVID-19 vaccination. Both unilateral [11–13] and bilateral [6, 7] cases of optic neuritis have been reported, and have occurred in patients with symptoms ranging from mild to severe. The most common presenting symptoms have included pain with eye movements, decreased visual acuity, and headache. This case was unique in that the patient did not have any visual symptoms, perhaps reflecting a subclinical or mild form of optic neuritis that did not impact visual acuity. However, she did exhibit pain with eye movement and optic nerve enhancement.

This patient was likewise unique in that she exhibited restrictions in extraocular motility, which one would not typically see in optic neuritis. Motility deficits have been seen in other inflammatory conditions of the eye that have been associated with COVID-19 infection, including orbital inflammatory pseudotumor and orbital apex syndrome, a syndrome characterized by dysfunction of the optic nerve (II), oculomotor nerve (III), trochlear nerve (IV), abducens nerve (VI), and the first division of the trigeminal nerve (V). Case reports of COVID-19 related orbital inflammatory disease or orbital apex syndrome have been rare [14, 15]. The most common symptoms in these cases have included vision loss, ptosis, periorbital pain, and ophthalmoplegia. Cases of COVID-19 related orbital apex syndrome have most often been associated with concurrent orbital cellulitis or mucormycosis coinfection, which our patient did not have. Moreover, orbital apex syndrome is commonly associated with enhancement in the orbital apex area on MRI, which was not seen in our patient. In our case, the patient’s restrictions in eye movement were limited to right eye supraduction, and may have been related to concomitant cranial nerve involvement, though less likely given her isolated deficit, or more likely, due to extraocular muscle involvement.

This is also the first case to the authors’ knowledge of optic neuritis occurring after *both* recent COVID infection and booster vaccination. Moreover, our patient began experiencing symptoms of pressure-like eye pain with eye movement within two hours after receiving her booster. This is much quicker than previously documented cases of COVID-19 related optic neuritis, which have occurred days to weeks after initial infection [12, 13], or several weeks after vaccination [8]. Though it is difficult to know precisely what led to this patient’s case of optic neuritis, COVID-19 infection, vaccination, or the combination of the two, the authors suspect that the combination of a recent COVID infection and booster vaccination may have contributed to the hyperacute presentation of her optic neuritis. The patient likely mounted an initial immune response, developing her own antibodies and memory B-cells against COVID-19 after contracting the virus, thus priming her against future exposures. Vaccination with the booster dose may have then triggered a rapid secondary inflammatory reaction that led to her optic neuritis. This is supported by studies demonstrating that COVID-19 recovered patients achieve a higher antibody and memory B-cell response to vaccination compared to COVID-19 naïve patients [16].

Moreover, it is well known that COVID-19 immunity is strongest initially after infection and wanes over time. Thus, the fact that she received her COVID-19 booster shortly after initial infection may have predisposed her to mounting a robust immune reaction, whereby viral antigens from the vaccine immediately triggered an immune response toward host myelin proteins in the central nervous system leading to optic neuritis. To date, there are no other reports of optic neuritis *or* other systemic immune reactions associated with COVID-19 vaccination after recent COVID-19 infection, and thus it is difficult to know for certain if this relationship is truly responsible for our patient’s presentation. The most recent recommendations by the Centres for Disease Control (CDC) allow for COVID-19 vaccination or booster vaccination as soon as 10 days after COVID-19 infection, which is much sooner than our patient received her COVID-19 booster following infection.

Ultimately, this case represents a rare neuro-ophthalmologic complication of COVID-19, and highlights the importance of considering SARS-CoV-2 infection in patients presenting with new onset visual and inflammatory ophthalmologic symptoms. High clinical suspicion is needed, and multidisciplinary evaluation including by the primary, neurology, and ophthalmology teams are required to determine appropriate treatment, often with high dose corticosteroids. Prognosis and visual recovery are typically good.

In conclusion, the patient presented in this study developed a hyperacute optic neuritis with a mild clinical presentation after receiving her COVID-19 booster vaccination. This case is unique in that the patient was exposed to both the virus and the vaccine prior to her presentation. Though it is difficult to know exactly what caused her presentation, it is possible that the initial exposure to the COVID-19 virus primed the patient’s immune system, leading to a rapid inflammatory response upon COVID vaccination. As our understanding of COVID-19 continues to grow, likely so will our knowledge of the various neuro-ophthalmological sequalae of SARS-CoV-2 infection.

## Data Availability

All relevant findings in the patient described here are included in this report.

## Abbreviations

APD: Afferent pupillary defect
OCT: Optical coherence tomography
HVF: Humphrey visual field
MRI: Magnetic resonance imaging
MS: Multiple sclerosis
